# Determination of the Optimal Bacterial DNA Extraction Method to Explore the Urinary Microbiota

**DOI:** 10.3390/ijms23031336

**Published:** 2022-01-25

**Authors:** Julie A. Vendrell, Steven Henry, Simon Cabello-Aguilar, Elise Heckendorn, Sylvain Godreuil, Jérôme Solassol

**Affiliations:** 1 Laboratoire de Biologie des Tumeurs Solides, Département de Pathologie et Oncobiologie, CHU Montpellier, Université de Montpellier, 34295 Montpellier, France; j-vendrell@chu-montpellier.fr (J.A.V.); s-cabelloaguilar@chu-montpellier.fr (S.C.-A.); eliseheckendorn@gmail.com (E.H.); 2 Laboratoire de Bactériologie, CHU Montpellier, Université de Montpellier, 34295 Montpellier, France; s-henry@chu-montpellier.fr (S.H.); s-godreuil@chu-montpellier.fr (S.G.); 3 UMR MIVEGEC, IRD-CNRS-Université de Montpellier, 34394 Montpellier, France; 4 Institut Régional du Cancer de Montpellier, ICM, 34298 Montpellier, France

**Keywords:** microbiota, DNA extraction, NGS

## Abstract

Recent advances in molecular biology have been successfully applied to the exploration of microbiota from various fluids. However, the urinary microbiota remains poorly explored, as its analysis requires specific technical considerations. Indeed, urine is a low microbial biomass environment, in which the representativity of each bacterium must be respected to obtain accurate data. Thus, sensitive extraction methods must be used to obtain good quality DNA while preserving the proportions between species. To address this, we compared the efficiency of five extraction methods on artificial urine samples spiked with low amounts of four bacteria species. The quality of the DNA obtained was further evaluated by different molecular biology approaches, including quantitative PCR and amplicon-based next-generation sequencing (NGS). Although two extraction methods allowed DNA of sufficient quality for NGS analysis to be obtained, one kit extracted a larger amount of DNA, which is more suitable for the detection of low-abundant bacteria. Results from the subsequent assessment of this kit on 29 human clinical samples correlated well with results obtained using conventional bacterial urine culture. We hope that our work will make investigators aware of the importance of challenging and adapting their practice in terms of the molecular biology approaches used for the exploration of microbiota.

## 1. Introduction

Until the recent discovery of a unique urinary microbiota in the urinary tract [1,2,3], urine was assumed to be a sterile environment in healthy people. Since the fall of this dogma, several studies have reported that the presence of specific bacteria might play a role in the maintenance of urinary health [2]. Some bacteria from the urinary commensal microbiota have recently been reported to promote/cause infections [4], urinary incontinence [5,6], kidney transplant rejection or dysfunction [6,7], and cancer development [8,9,10].

Standard urine cultures have long been used in clinical microbiology laboratories to detect uropathogens, especially during a urinary tract infection (UTI) episode. However, these analyses, which allow the detection of viable bacteria, also exhibit several important limitations: they are less adapted to the detection of anaerobic and slowly growing microorganisms and do not allow the detection of unknown pathogens. Although there have been recent improvements to urine culture techniques, particularly modification of culture media and identification of isolated species by matrix-assisted laser desorption ionization–time of flight mass spectrometry [5], these semiquantitative techniques are not fully adapted for the characterization of complex and relatively low-abundant microbial mixtures or to highlight dysbiosis. Recently, the development of molecular approaches using next-generation sequencing (NGS) has circumvented these limitations. Indeed, amplicon-based sequencing of the hypervariable regions of the 16S rRNA genes and shotgun sequencing enables the determination of bacterial species’ composition within complex fluids and the detection of non-cultivable bacteria [11,12,13].

However, for molecular tools, and especially sequencing techniques, DNA extraction is a critical step for obtaining accurate data. Indeed, in the case of the extensively studied gut microbiota, the extraction technique employed to extract DNA has been shown to impact results [14,15]. A large number of studies have benchmarked several DNA extractions kits for analysis of microbiota from various types of samples, such as feces, blood, or vaginal fluid [16,17,18,19]. However, the urinary microbiome has been less explored. Assessment of the urinary microbiota is highly challenging, as it is composed of low biomass communities and is less abundant and diverse than other microbiota. Only one recent study has evaluated a limited number of different available DNA extraction kits to analyze the microbiota of human urine samples [20]. In this work, five commercial DNA extraction methods were benchmarked using 11 human urine samples, and two kits gave the best performances. To complement this work and validate it on another sample cohort, we compared the efficiency of these two kits and three newly available commercial methods to extract bacterial DNA from well-characterized artificial urine and human clinical samples and evaluated the quality of the DNA obtained by performing different molecular biology approaches, including amplicon-based NGS.

## 2. Results

### 2.1. Quantification of DNA Extracted from Artificial Urine Samples

**Figure 1:**
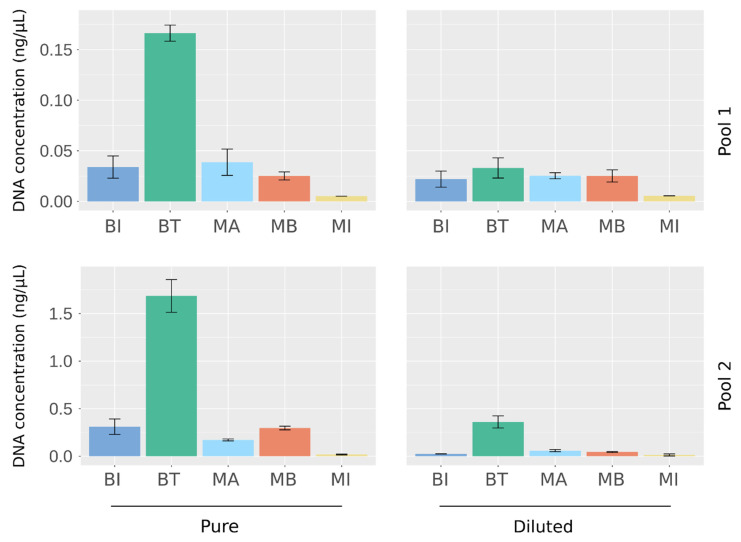
Quantification of DNA extracted from artificial urine samples using the five DNA extraction kits. DNA concentration was measured by fluorimetry (Qubit). Results are means ± SD of the value obtained for the extraction triplicates.

To select the best extraction method for analysis of the microbiome of urine samples, we used a large volume of sterilized urine that we spiked with four species of bacteria at different abundances: *Lactobacillus delbrueckii* and *Enterococcus faecalis* were spiked at a high abundance, *Prevotella bivia* and *Escherichia coli* at a low abundance. Two separate pools, Pool 1 and Pool 2, were generated and then diluted at 1:10 or 1:20, respectively, to evaluate the performance of the kits when there were different amounts of bacteria in the sample. Equal volumes of the four established pools were subjected to parallel extraction with five commercial methods (Table 1). To assess reproducibility, each experimental condition was assessed in triplicate. The total DNA recovered was firstly quantified by fluorimetry, which revealed differences between the kits: the BT kit obtained the highest DNA yield, and the MI kit obtained the lowest, irrespective of the conditions tested (Figure 1). Similar DNA quantities were extracted using the BI, MB, and MA kits.

**Figure 2:**
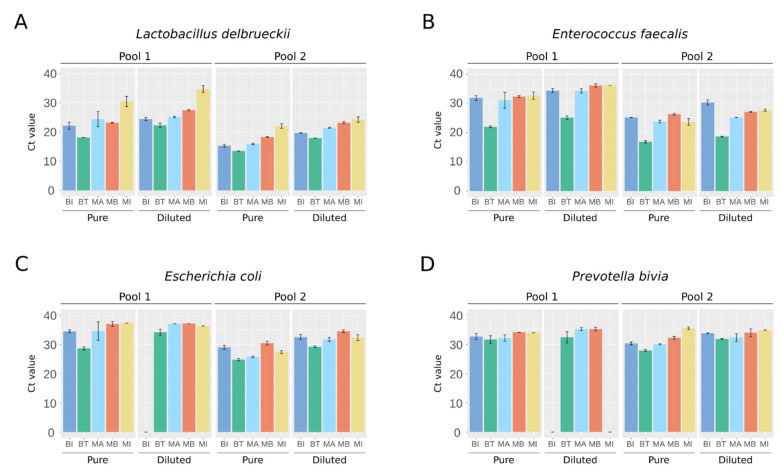
Quantitative PCR measurements specific to the bacteria spiked in the artificial urine samples. Ct value for: (**A**) *L. delbrueckii*, (**B**) *E. faecalis*, (**C**) *E. coli*, and (**D**) *P. bivia*. Results are means ± SD of the Ct values obtained for the extraction triplicates. Values representative of two independent qPCR experiments.

As a high level of total DNA quantified could reflect contamination by human host DNA, we performed quantitative PCR (qPCR) experiments using primers specific to each spiked bacterium. Whatever the bacteria and their abundance in the mixture, the BT kit resulted in the lowest cycle threshold (Ct) value, meaning that it recovered the highest amount of DNA (Figure 2). For the two bacteria species spiked at the lowest abundance, no signal was detected in Pool 1 diluted at 1:10 by the BI kit for *E. coli* (Figure 2C) and by the BI and MI kits for *P. bivia* (Figure 2D). The amounts of DNA obtained in the extraction triplicates were relatively homogeneous per bacteria species, highlighting that the different kits all had satisfactory reproducibility (Figure 2).

Overall, our results indicate that the BT kit seems able to recover more DNA than the other kits tested, regardless of the bacteria and their representativity in a mixture.

### 2.2. NGS Detection of Bacteria Present in Artificial Urine Samples

**Figure 3:**
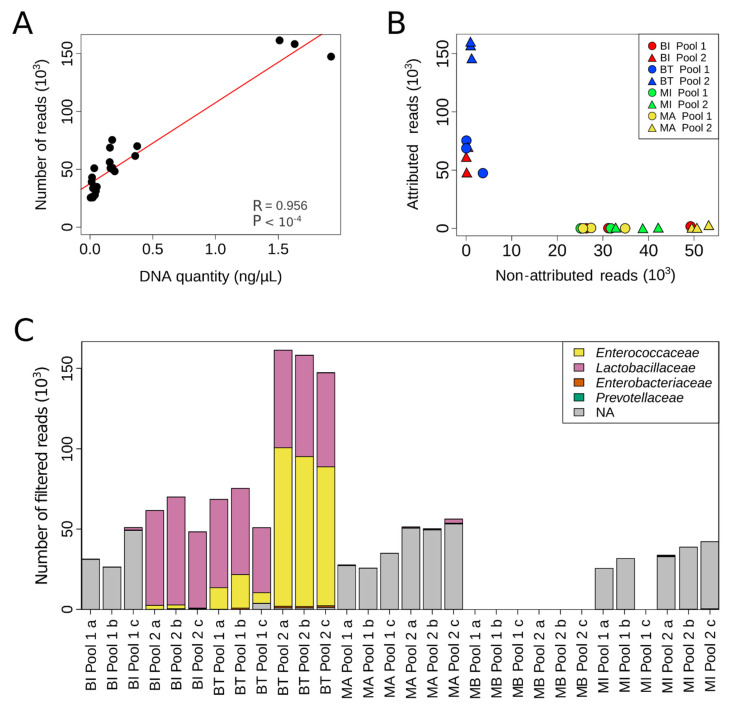
Microbial composition of the artificial urine samples assessed by NGS. (**A**) Correlation between the quantity of DNA extracted and the total number of reads obtained (R, Pearson’s correlation test). (**B**) Per sample analyzed, representation of the number of reads non-attributed to an OTU in relation to the number of reads attributed to an OTU. (**C**) Taxonomic assignment of the reads. In gray (NA), reads are not assigned to a family.

To obtain more in-depth information, we decided to use a specific NGS protocol that allowed the sequencing of eight of nine hypervariable regions of the 16S rRNA gene. However, this requires the generation of long reads (2 × 300 bp) with a quality score >Q30. Since the pool samples diluted at 1:10 or 1:20 were not concentrated enough to be analyzed using this NGS approach, only non-diluted pool samples were used (data not shown). Bioinformatics analysis of the NGS experiments revealed a significant correlation between the number of filtered reads and the amount of DNA extracted (Figure 3A; *p* < 1 × 10^−4^, Pearson’s correlation test). Next, we evaluated whether the quality of DNA extracted could influence taxonomic assignment. Interestingly, a large proportion of reads generated from DNA extracted from the most concentrated extract (Pool 2) with the BI kit, and from DNA extracted from both Pool 1 and Pool 2 with the BT kit, could be attributed to an operational taxonomic unit (OTU) (Figure 3B). For the other samples, more than 95% of reads were not attributed because they were not of sufficient quality to allow taxonomic determination (Figure 3B and Table S1). Of particular interest, reads obtained with the BT kit were more informative, as a similar number of reads could be attributed to an OTU from Pool 1 extracted with the BT kit as from Pool 2 extracted with the BI kit, despite the fact that Pool 1 contains half the amount of DNA of Pool 2 (mean: 0.33 and 0.62 ng, respectively; Figure 3B).

We then analyzed the different taxa present in and their representativity within the pools (Figure 3C, Table S1). The four spiked bacteria were only detected in the Pool 2 triplicates extracted with the BT kit. Moreover, bacteria proportions were respected in accordance with the known abundance of the spiked bacteria and the qPCR results previously presented (Figure 3C and Table S1). For DNA extracted from Pool 1 with the BT kit and from Pool 2 with the BI kit, the bacteria spiked at the lowest abundance were detectable in some samples but not in all triplicates. For the other extractions (BI kit Pool 1, MI and MA kits Pool 1 and Pool 2), the percentage of non-attributed reads was too great and their quality too low to allow taxonomic determination. Altogether, DNA extraction with the BT kit appears better than extraction by other kits for subsequent NGS or qPCR analysis of samples from relatively low microbial biomass environments.

### 2.3. Evaluation of the BT Kit’s Performance on Human Clinical Samples

We further evaluated the ability of the BT kit to detect the different types of bacteria present in complex human specimens by use of human clinical urine samples. Their infectious status had been previously determined using conventional bacterial urine culture. For this purpose, 8 mL of urine samples obtained from 16 healthy patients (HP) and 13 patients with UTIs caused by *E. coli* were collected and DNA was extracted with the BT kit. The level of *E. coli* DNA in the samples was firstly measured by qPCR. No analyzable signal (>37 Ct) was detected for the 16 HP samples, whereas a clear signal was obtained for the 13 UTI samples, with Ct values ranging from 7.85 to 34.82 (Table S2). Interestingly, the Ct values obtained inversely correlated with the colony forming unit (CFU) values measured by conventional bacterial urine culture (n = 13, *p* < 10^−3^, r = −0.80, Spearman’s rank correlation test), underscoring the accuracy of the BT kit for DNA extraction from urine samples.

**Figure 4:**
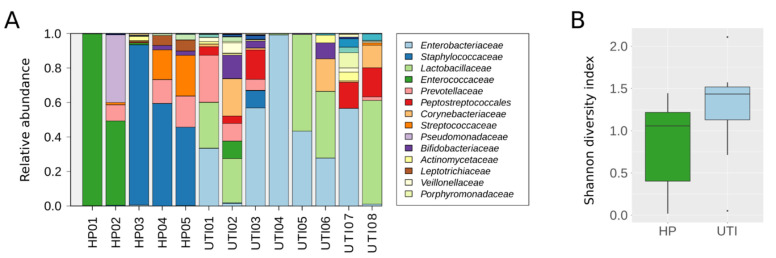
Microbial composition of 13 human clinical samples extracted using the BT kit. (**A**) Bacterial relative abundance by family. Only the 14 most abundant families could be visualized on the histograms. (**B**) Shannon diversity index. Box plots depict the median and range of diversity measures. HP, DNA extracted from urine samples from healthy patients; UTI, DNA extracted from urine samples from patients with urinary tract infections.

We further analyzed five HP samples and eight UTI samples by NGS to evaluate whether the recovered DNA was of sufficient quality to obtain satisfactory results and allow taxonomic analysis. After sequencing, a mean of 111,301 raw reads were obtained per sample. After quality filtering, primer trimming, and merging pairs, reads were assigned to OTUs and corresponded to 64 different families. However, as the aim of our study was not to focus on rare families, we preserved for further analysis the 34 families that had an abundance of up to 0.1% in at least one sample (Figure 4A, Table S3). As expected, the most prevalent family detected in the eight UTI samples was *Enterobacteriaceae*, with a high proportion of attributed reads. Moreover, the number of assigned reads per sample significantly correlated with the CFU measured by conventional bacterial urine culture (n = 8; *p* = 0.007, r = 0.85, Spearman’s rank correlation test). In addition, the families detected differed between the two group of samples. The *Enterococcaceae* and *Streptococcaceae* families were the most prevalent (3/5 and 4/5 samples, respectively) and abundant families in HP samples, while they were scarce in UTI samples (1/8 and 2/8, respectively). Conversely, the *Lactobacillaceae* family was highly prevalent in UTI samples (7/8) but was almost completely absent in HP samples (1/5). Moreover, when we measured the microbial diversity in the 13 samples analyzed, we found no significant difference in the Shannon diversity index between the HP and UTI samples, although this is likely to be because of the low number of samples analyzed per group, as a tendency towards significance was observed (*p* = 0.12, Mann–Whitney U test, Figure 4B).

## 3. Discussion

Despite the latest advances in molecular biology, the urinary microbiota still remains a poorly explored field of investigation, as working with this type of fluid is quite challenging for various reasons: (i) urine is composed of a very low microbial biomass compared with other types of samples; (ii) samples have different salt concentrations that may disturb DNA extraction [21]; and (iii) the presence of PCR inhibitors in urine may further affect bacteria identification assays [22]. Moreover, Gram-positive bacteria and mycobacteria can be more difficult to lyse than Gram-negative bacteria due to their specific envelope structure, which affects their representativity in a complex microbial community [23]. To select the optimal DNA extraction method that is well-suited to this particular matrix, we evaluated five available commercial methods on urine samples spiked with Gram-positive and Gram-negative bacteria. For these artificial samples, bacteria were spiked at very low quantities to mimic the difficult conditions that can be encountered when working with clinical samples. Moreover, each artificial sample was extracted in triplicate and further explored by qPCR and NGS. Using these artificial samples, the highest yield was obtained with the BT kit and the lowest with the MI kit. However, reproducibility was satisfactory for all tested kits. All kits allowed the consistent detection of the four bacteria, with differences in the levels of detection. However, when these samples were further explored by NGS, which requires high-quality DNA, relevant results were only obtained with the BT kit on Pool 2. Indeed, it was the only experimental condition under which the four spiked bacteria were detected with respect to their representativeness. The BI kit on Pool 2 and BT kit on Pool 1 detected the most abundant bacteria but missed the bacteria at the lowest abundance, certainly due to the low DNA amounts used in the reactions. For the other kits (MI, MB, MA), the number of non-attributed reads were too high to allow any accurate taxonomic assignment, suggesting they are unable to obtain sufficient quantity and/or quality of DNA for NGS assays. As NGS experiments require larger quantities of DNA than qPCR, extraction methods that allow a high yield of DNA extraction are more suitable for this type of application.

Illustrating the importance of the extraction step in an analytical process, two very recent studies have also benchmarked DNA extraction kits for the study of human and canine urine microbiota [20,24]. Mrofchak et al. identified the BI kit as having the best extraction method for the study of the urine microbiota, whereas the study by Karstens et al. reported that the BI and BT kits gave similar performances. In these studies, authors benchmarked the kits using patient samples, for which the microbiota composition was unknown. In our work, we furthered their investigations by evaluating other available methods of extraction on well-defined artificial samples. In our hands, the best results were obtained with the BT kit, particularly for exploration of the microbiota using NGS approaches (the BI kit did give relevant results but required larger volumes of urine to obtain similar amounts of DNA). Another point that we did not evaluate in this study and that should be carefully taken into consideration is the urine sampling method used, which has recently been reported to influence urine microbiota composition [25,26].

We evaluated the BT extraction method further by use of 29 clinical samples previously characterized by conventional microbacterial techniques. Interestingly, the results obtained by qPCR and NGS correlated with those obtained with culture techniques. Indeed, the amount of *E. coli* detected by molecular approaches correlated highly with the results obtained by bacterial urine culture. Other bacteria revealed by culture approaches were also highlighted by NGS exploration, underscoring the relevance of the extraction method to specifically explore the urinary microbiota. Finally, use of the BT extraction method resulted in sequencing reads of high quality compared with other studies: Gottschick et al. reported that 46% of the reads passed quality filters and 20% were assigned at the family level [27] whereas in our experiments, 61.5% of the sequencing reads obtained were correctly assigned at the family level. This discrepancy could be of upmost importance if we were interested in species that were poorly represented in the urine microbiota. However, these results were obtained on a small number of samples and thus need to be validated on a larger sample cohort.

Recent analyses using molecular techniques have shown that knowing the overall composition of microbiomes is important for health and disease comprehension. However, a fundamental challenge in these studies is the isolation of DNA representative of the entire resident microbial community. As such, this requires specific technical considerations to account for issues that might arise owing to a low microbial biomass environment such as urine. Notably, one should select the best extraction method according to the samples analyzed, the molecular biology approaches used, and the technical level of sensitivity required. We hope that our work will make investigators aware of the importance of challenging and adapting their practice to minimize bias and obtain informative results.

## 4. Materials and Methods

### 4.1. Artificial Sample Preparation

Urine from a healthy male volunteer was sterilized by ultrafiltration with a 0.2 µm asymmetric polyethersulfone membrane filter (Thermo Fischer Scientific, Illkirch-Graffenstaden, Germany). The absence of bacteria was tested by plating 200 µL both on Columbia agar with 5% sheep blood and Chocolate agar (Becton-Dickinson, Le Pont-de-Claix, France). These were incubated at 35 °C for 4 days under aerobic and anaerobic standard conditions. The sterilized urine was then separated into 2 extracts, which were spiked with 4 bacteria at different abundances: *L. delbrueckii* (Gram-positive) obtained from clinical samples and *E. faecalis* (Gram-positive) obtained from ATCC 29212 at a high abundance; *P. bivia*, (Gram-negative) obtained from clinical samples and *E. coli* (Gram-negative) obtained from ATCC 25922 at a low abundance. The first extract (called Pool 1) was used pure or diluted at 1:10 and the second (called Pool 2) pure or diluted at 1:20.

### 4.2. DNA Extraction and Total Quantification

1 mL of each extract was centrifuged at 5000× *g* for 10 min at 4 °C, and the pellet was subjected to DNA extraction using five commercial methods. Each condition was performed in triplicate. The methods used were: (1) QIAmp BiOstic Bacteremia kit (BI, Qiagen, Hilden, Germany); (2) QIAmp DNA Microbiome kit (MI, Qiagen); (3) AMPure XP (MB, Beckman-Coulter, Brea, CA, USA); (4) DNeasy Blood and Tissue kit (BT, Qiagen); (5) MagNA Pure Compact Kit (MA, Roche, Meylan, France) (Table 1). Manufacturers’ protocols were performed with the following details: for the BT kit, the Gram-positive optional steps were performed; for the MA kit, all the optional lysis steps were performed, including the addition of Bacterial Lysis Buffer (Roche), enzymatic cocktail, and proteinase K, and the boiling step; for the MB kit, the same optional lysis steps as for the MA kit were performed. For all kits, DNA was eluted in 50 µL of elution buffer or water, as per the manufacturers’ recommendations. For each condition, extracted DNA was quantified on a Qubit 2.0 fluorometer with the Qubit dsDNA HS Assay Kit (Thermo Fisher Scientific, Waltham, MA, USA).

### 4.3. Human Sample Collection

Midstream urine samples from healthy patients or patients with UTI were collected. DNA from these samples were extracted with the Qiagen DNeasy Blood and Tissue kit (BT kit).

### 4.4. Quantitative PCR

The resulting extracted DNA, from both artificial and clinical samples, underwent qPCR using the SYBR Green I Master (Roche) in combination with a LightCycler 480 Instrument (Roche). Specific primers were validated on pure boiled bacteria (Table S4). Results were expressed as the Ct value, which was defined as the number of cycles required for the fluorescent signal to cross the threshold. Ct values were inversely proportional to the amount of targeted nucleic acid in the sample.

### 4.5. Next-Generation Sequencing Experiments

The samples underwent a PCR amplifying the V1–V3, V3–V4, V4–V5, and V6–V8 regions of the 16S rRNA gene (Table S4). Regions of interest were amplified using the Access Array (Fluidigm, San Francisco, CA, USA) in combination with a 48.48 Fluidigm Access Array System. Libraries were then collected, indexed, pooled, qualified by use of D1000 ScreenTapes and a 4200 TapeStation instrument (Agilent Technologies, Santa Clara, CA, USA), and quantified using the KAPA Library Quantification Kit (Roche) on a LightCycler 480 (Roche). Pair-end sequencing (2 × 300 cycles) was finally performed using a MiSeq instrument (Illumina, San Diego, CA, USA).

### 4.6. Bioinformatics Analysis

Raw sequences were processed and filtered with DADA2 (v1.16) on Rstudio [28]. Briefly, primers were cut using the cutadapt function, and reads with a quality score lower than 30 were truncated using the filterAndTrim function. The paired forward and reverse reads were then merged, and the sequences were compared to the SILVA reference database using the assignTaxonomy function [29].

### 4.7. Statistical Analysis

Statistical analysis was carried out using Rstudio. Spearman’s rank correlation test, Pearson’s correlation test, and the Mann–Whitney U test were considered to be statistically significant if *p*-values were less than 0.05.

## Figures and Tables

**Table 1:** Characteristics of extraction kits evaluated.

Kit	Full Name	Supplier	Lysis Method				Extraction Method	DNA Binding Principle
			Chemical	Enzymatic	Mecanical	Heat		
BI	QIAmp BiOstic Bacteremia	Qiagen	Yes	PK	Yes	70 °C	Manual	Silica spin-column
BT	DNeasy Blood and Tissue	Qiagen	Yes	PK and Ly	No	No	Manual	Silica spin-column
MA	MagNA Pure Compact	Roche	Yes	PK and Ly	No	95 °C	Automated	Magnetic beads
MB	AMPure XP Beads	Beckman-Coulter	Yes	PK and Ly	No	95 °C	Manual	Magnetic beads
MI	QIAmp DNA Microbiome	Qiagen	Yes	PK	Yes	No	Manual	Silica spin-column

PK, proteinase K; Ly, lysozyme.

## Data Availability

The data presented in this study are available in the present article or in the supplementary materials.

## Supplementary Materials

The following information can be downloaded at: https://www.mdpi.com/article/10.3390/ijms23031336/s1.
