# Supplementary material for: Determination of the Optimal Bacterial DNA Extraction Method to Explore the Urinary Microbiota
Source: Int J Mol Sci. 2022 Jan 25;23(3):1336. doi: 10.3390/ijms23031336 (PMC8835916; doi:10.3390/ijms23031336)
Supplement: Supplementary file 1 [file ijms-23-01336-s001.zip › ijms-1548482-supplementary.pdf]

**Table S1.** Number of reads attributed to an OTU by NGS analysis for the artificial urine samples

| Sample | *Enterobacteriaceae* | *Enterococcaceae* | *Lactobacillaceae* | *Prevotellaceae* | Non-attributed reads |
|---|---|---|---|---|---|
| BI Pool 1a | 0 | 103 | 58 | 0 | 31188 |
| BI Pool 1b | 9 | 0 | 53 | 0 | 26360 |
| BI Pool 1c | 184 | 0 | 1530 | 2 | 49229 |
| BI Pool 2a | 11 | 2533 | 58929 | 31 | 28 |
| BI Pool 2b | 404 | 2334 | 67257 | 0 | 20 |
| BI Pool 2c | 420 | 275 | 47447 | 0 | 136 |
| MI Pool 1a | 0 | 0 | 0 | 0 | 25531 |
| MI Pool 1b | 0 | 0 | 0 | 0 | 31745 |
| MI Pool 2a | 497 | 317 | 0 | 0 | 32846 |
| MI Pool 2b | 214 | 47 | 3 | 0 | 38777 |
| MI Pool 2c | 171 | 338 | 294 | 0 | 42167 |
| BT Pool 1a | 134 | 13345 | 55056 | 56 | 12 |
| BT Pool 1b | 771 | 20858 | 53727 | 0 | 42 |
| BT Pool 1c | 125 | 6648 | 40452 | 0 | 3654 |
| BT Pool 2a | 999 | 98739 | 60597 | 37 | 976 |
| BT Pool 2b | 825 | 93323 | 62995 | 9 | 981 |
| BT Pool 2c | 1020 | 86499 | 58418 | 168 | 1233 |
| MA Pool 1a | 0 | 0 | 329 | 0 | 27406 |
| MA Pool 1b | 0 | 0 | 0 | 0 | 25619 |
| MA Pool 1c | 0 | 0 | 42 | 0 | 34871 |
| MA Pool 2a | 0 | 32 | 579 | 0 | 50682 |
| MA Pool 2b | 39 | 46 | 609 | 0 | 49536 |
| MA Pool 2c | 435 | 0 | 2517 | 0 | 53248 |

OTU, operational taxonomic unit; NGS, next-generation sequencing.

**Table S2.** Detection of *E. Coli* by qPCR in 29 human clinical samples previously characterized by conventional bacterial urine culture.

| Sample | qPCR (Ct value) | Conventional bacterial urine culture (CFU) |
|---|---|---|
| HP01 | >37 | N.D. |
| HP02 | >37 | N.D. |
| HP03 | >37 | N.D. |
| HP04 | >37 | N.D. |
| HP05 | >37 | N.D. |
| HP06 | >37 | N.D. |
| HP07 | >37 | N.D. |
| HP08 | >37 | N.D. |
| HP09 | >37 | N.D. |
| HP10 | >37 | N.D. |
| HP11 | >37 | N.D. |
| HP12 | >37 | N.D. |
| HP13 | >37 | N.D. |
| HP14 | >37 | N.D. |
| HP15 | >37 | N.D. |
| HP16 | >37 | N.D. |
| UTI01 | 30.31 | 1000 |
| UTI02 | 32.26 | 1000 |
| UTI03 | 32.52 | 10000 |
| UTI04 | 12.35 | 1000000 |
| UTI05 | 20.04 | 1000000 |
| UTI06 | 29.49 | 10000 |
| UTI07 | 21.95 | 100000 |
| UTI08 | 33.63 | 10000 |
| UTI09 | 34.82 | 5000 |
| UTI10 | 7.85 | 1000000 |
| UTI11 | 15.06 | 10000000 |
| UTI12 | 18.82 | 10000000 |
| UTI13 | 10.83 | 10000000 |

qPCR, quantitative PCR; Ct, cycle threshold; CFU, Colony forming unit; N.D., not detectable.

**Table S3.** Number of filtered reads assigned to an OTU by NGS analysis for the human clinical samples

| | Healthy patient samples | | | | | Samples from patients with a urinary tract infection | | | | | | | |
|---|---|---|---|---|---|---|---|---|---|---|---|---|---|
| Family | HP01 | HP02 | HP03 | HP04 | HP05 | UTI01 | UTI02 | UTI03 | UTI04 | UTI05 | UTI06 | UTI07 | UTI08 |
| Non-attributed reads | 741 | 27317 | 68468 | 15239 | 94858 | 93459 | 86678 | 80608 | 8163 | 1111 | 31055 | 11268 | 12661 |
| Actinomycetaceae | 0 | 0 | 932 | 2 | 0 | 315 | 450 | 232 | 0 | 0 | 1447 | 2304 | 0 |
| Aerococcaceae | 0 | 0 | 0 | 0 | 0 | 350 | 109 | 15 | 0 | 0 | 0 | 1460 | 191 |
| Anaerovoracaceae | 0 | 0 | 0 | 0 | 0 | 0 | 96 | 0 | 0 | 0 | 0 | 61 | 0 |
| Atopobiaceae | 0 | 0 | 0 | 1209 | 477 | 0 | 1238 | 9 | 0 | 0 | 0 | 0 | 0 |
| Bacteroidaceae | 0 | 0 | 128 | 0 | 0 | 15 | 0 | 0 | 0 | 0 | 0 | 0 | 0 |
| Bifidobacteriaceae | 0 | 0 | 15 | 2806 | 386 | 14 | 6609 | 1359 | 0 | 306 | 2961 | 0 | 0 |
| Campylobacteraceae | 0 | 0 | 0 | 0 | 0 | 0 | 39 | 0 | 0 | 0 | 0 | 172 | 0 |
| Carnobacteriaceae | 131 | 684 | 0 | 0 | 6 | 66 | 9 | 186 | 89 | 0 | 2 | 0 | 0 |
| Cladosporiaceae | 0 | 0 | 49 | 0 | 0 | 0 | 0 | 0 | 0 | 0 | 0 | 0 | 0 |
| Corynebacteriaceae | 0 | 0 | 195 | 45 | 0 | 330 | 10424 | 346 | 30 | 0 | 6073 | 345 | 5163 |
| Debaryomycetaceae | 0 | 0 | 0 | 7 | 24 | 33 | 0 | 0 | 0 | 0 | 0 | 0 | 0 |
| Enterobacteriaceae | 26 | 0 | 12 | 27 | 0 | 7386 | 719 | 18584 | 197203 | 26256 | 8946 | 25408 | 427 |
| Enterococcaceae | 177540 | 45627 | 385 | 33 | 0 | 27 | 4890 | 7 | 39 | 0 | 0 | 0 | 0 |
| Fusobacteriaceae | 0 | 0 | 6 | 0 | 0 | 0 | 12 | 0 | 0 | 0 | 0 | 2216 | 0 |
| Hungateiclostridiaceae | 0 | 0 | 0 | 0 | 0 | 0 | 128 | 22 | 0 | 0 | 0 | 0 | 0 |
| Lachnospiraceae | 0 | 0 | 15 | 0 | 59 | 15 | 27 | 30 | 0 | 0 | 274 | 0 | 1485 |
| Lactobacillaceae | 204 | 0 | 0 | 12 | 0 | 5883 | 12427 | 73 | 1495 | 34024 | 12452 | 0 | 23648 |
| Leptotrichiaceae | 0 | 0 | 0 | 6359 | 955 | 0 | 75 | 7 | 0 | 0 | 0 | 0 | 0 |
| Mycoplasmataceae | 0 | 0 | 0 | 99 | 8 | 0 | 502 | 673 | 0 | 0 | 0 | 0 | 0 |
| Peptococcaceae | 0 | 0 | 0 | 0 | 0 | 0 | 69 | 0 | 0 | 0 | 0 | 0 | 0 |
| Peptostreptococcaceae | 0 | 0 | 0 | 0 | 0 | 0 | 121 | 0 | 0 | 0 | 0 | 0 | 0 |
| Peptostreptococcales-Tissierellales_fa | 0 | 0 | 173 | 154 | 14 | 1119 | 2099 | 5575 | 6 | 5 | 0 | 6770 | 6615 |
| Porphyromonadaceae | 0 | 0 | 0 | 0 | 0 | 25 | 416 | 0 | 0 | 0 | 0 | 3946 | 0 |
| Prevotellaceae | 1 | 8733 | 0 | 15299 | 2708 | 6011 | 4950 | 2072 | 0 | 0 | 0 | 8 | 824 |
| Propionibacteriaceae | 0 | 0 | 149 | 1 | 9 | 34 | 15 | 33 | 0 | 0 | 11 | 405 | 75 |
| Pseudomonadaceae | 0 | 36628 | 8 | 0 | 0 | 7 | 0 | 0 | 0 | 0 | 0 | 0 | 0 |
| Pseudonocardiaceae | 0 | 0 | 1 | 0 | 0 | 0 | 134 | 0 | 0 | 0 | 0 | 0 | 0 |
| Rhodanobacteraceae | 3 | 0 | 256 | 0 | 0 | 8 | 32 | 189 | 0 | 0 | 0 | 0 | 0 |
| Ruminococcaceae | 0 | 0 | 0 | 0 | 0 | 0 | 0 | 0 | 0 | 0 | 14 | 704 | 0 |
| Staphylococcaceae | 0 | 392 | 34463 | 66256 | 6834 | 36 | 160 | 3257 | 0 | 0 | 0 | 0 | 0 |
| Streptococcaceae | 0 | 1182 | 108 | 19173 | 3530 | 0 | 63 | 0 | 2 | 0 | 0 | 0 | 551 |
| Sutterellaceae | 0 | 0 | 0 | 0 | 0 | 0 | 132 | 0 | 0 | 0 | 0 | 0 | 0 |
| Tannerellaceae | 0 | 0 | 0 | 0 | 0 | 0 | 133 | 0 | 0 | 0 | 0 | 0 | 0 |
| Veillonellaceae | 0 | 28 | 46 | 56 | 10 | 515 | 2885 | 3 | 0 | 0 | 0 | 1086 | 367 |
| Total number of reads | 178646 | 120591 | 105409 | 126777 | 109878 | 115648 | 135641 | 113280 | 207027 | 61702 | 63235 | 56153 | 52007 |

Numbers highlighted in red represented families with an abundance of up to 0.1% in the considered sample. OTU, operational taxonomic unit; NGS, next-generation sequencing.

**Table S4.** Primer sequences used in qPCR and NGS experiments

| Forward primers | Reverse primers | Use | Reference |
|---|---|---|---|
| ACACTGACGACATGGTTCTACAAGAGTTTGATCMTGGCTCAG | TACGGTAGCAGAGACTTGGTCTGWATTACCGCGGCKGCTG | V1-V3 exploration by NGS | *Weisburg et al., 1991* |
| ACACTGACGACATGGTTCTACACCTACGGGNGGCWGCAG | TACGGTAGCAGAGACTTGGTCTGGACTACNVGGGTWTCTAAT | V3-V4 exploration by NGS | *Chen et al., 2019* |
| ACACTGACGACATGGTTCTACAGTGCCAGCMGCCGCGGTAA | TACGGTAGCAGAGACTTGGTCTCCGYCAATTYMTTTRAGTTT | V4-V5 exploration by NGS | *Carporaso et al., 2010* |
| ACACTGACGACATGGTTCTACAAAACTYAAAKGAATTGACGG | TACGGTAGCAGAGACTTGGTCTACGGGCGGTGTGTRC | V6-V8 exploration by NGS | *Engelbrektson et al., 2010* |
| TGGTGATTACCGACGAAAACGGC | ACGCGTGGTTACAGTCTTGCG | qPCR *E. coli* | *Bej et al., 1991* |
| GGGATGCGTCTGATTAGCTTGTT | CTGCACGCTACTTGGCTGGTTC | qPCR *P. bivia* | *Zozaya-Hinchliffe et al., 2010* |
| GCCCTAAAGACTGGGATACCACT | CATCATTGCCTTGGTAGGCCTT | qPCR *L. delbrueckii* | Home-designed |
| CCCGAGTGCTTGCACTCAAT | GGGACGTTCAGTTACTAACGTCCT | qPCR *E. faecalis* | Home-designed |

qPCR, quantitative PCR; NGS, next-generation sequencing.
